# Prenatal and Neonatal Characteristics of Children with Prader-Willi Syndrome

**DOI:** 10.3390/jcm11030679

**Published:** 2022-01-28

**Authors:** Lionne N. Grootjen, Nathalie E. M. Uyl, Inge A. L. P. van Beijsterveldt, Layla Damen, Gerthe F. Kerkhof, Anita C. S. Hokken-Koelega

**Affiliations:** 1 Dutch Reference Center for Prader-Willi Syndrome Westzeedijk 106, 3016 AH Rotterdam, The Netherlands; l.damen@kindengroei.nl (L.D.); g.kerkhof@erasmusmc.nl (G.F.K.); a.hokken@erasmusmc.nl (A.C.S.H.-K.); 2 Department of Pediatrics, Subdivision of Endocrinology, Erasmus University Medical Center-Sophia Children’s Hospital, 3015 CN Rotterdam, The Netherlands; i.vanbeijsterveldt@erasmusmc.nl; 3 Dutch Growth Research Foundation, 3016 AH Rotterdam, The Netherlands; n.uyl@kindengroei.nl

**Keywords:** Prader-Willi Syndrome, neonate, prenatal, children

## Abstract

Objective: Prader-Willi syndrome (PWS) is a rare genetic syndrome with a wide spectrum of clinical features in early life. Late diagnoses are still present. We characterized the perinatal and neonatal features of PWS, compared them with those of healthy newborns and assessed the prenatal and neonatal differences between the genetic subtypes. Design: A cohort study in children with PWS. The prevalence of variables was compared with healthy infants (PLUTO cohort) and to population statistics from literature. Patients: 244 infants with PWS and 365 healthy infants. Measurements: Data on prenatal and neonatal variables in both cohorts. Population statistics were collected through an extensive literature search. Results: A higher prevalence of maternal age >35 years was found in PWS compared to healthy infants and population statistics, and the highest maternal age was found in the mUPD group. Higher prevalence of polyhydramnios, caesarean section, labour induction and breech presentation, and lower birth weight SDS was found in PWS compared to healthy infants. High prevalences of decreased fetal movements (78.5%), hypotonia (100%), cryptorchism (95.9%) and poor sucking/tube feeding (93.9%) were found in PWS. Conclusions: This study presents an overview of prenatal and neonatal variables in infants with PWS compared to healthy infants. Our findings may increase clinical awareness of the early perinatal signs of PWS by obstetricians, neonatologists and all those involved in infant care, enabling early diagnosis and start of multidisciplinary treatment.

## 1. Introduction

Prader-Willi syndrome (PWS) is a rare and complex genetic syndrome characterized by hypotonia, infantile feeding difficulties and, later on, hyperphagia, which will lead to obesity when uncontrolled, combined with many comorbidities such as short stature, typical facial dysmorphism, psychomotor delay, hypogonadism, behavioral abnormalities and cognitive impairment [1,2]. Hypothalamic dysfunction can explain most of the symptoms of PWS [3]. The prevalence of PWS varies across countries, but an incidence of 1 in 10.000–30.000 newborns is often reported [1,4,5].

PWS is caused by a lack of expression of the 15q11-q13 region on the paternally inherited chromosome, caused by a deletion (65–75%), maternal uniparental disomy (mUPD) (20–30%), imprinting center defect (ICD) (1–3%), or a translocation (<1%) [1].

Despite the availability of advanced genetic testing that makes it possible to diagnose children in the first few months of life, delayed diagnosis of PWS is still reported [6,7]. This might be related to the inadequate knowledge of physicians about PWS clinical features in early life. In contrast to neonates, the features of PWS are evident and more distinguishable in later life. Symptoms and dysmorphic features of PWS may be subtle in early life or symptoms may be masked by neonatal complications, making it difficult for physicians to recognize PWS [8]. Early diagnosis is important as it allows an early start of adequate multidisciplinary care and recombinant human growth hormone (rhGH) treatment. rhGH improves the abnormal body composition and has positive effects on cognition, mental and motor development [9,10,11,12,13]. An earlier start of rhGH treatment has positive effects on height and metabolic parameters [14], stabilizing BMI [15] and was associated with an earlier start of walking [16]. Together, multidisciplinary treatment and an early start of rhGH may prevent obesity and its consequences in the future [17]. It is, therefore, important to increase physician’s awareness of the maternal, perinatal and neonatal features of PWS.

Although perinatal and neonatal characteristics in PWS have been reported [6,18,19,20,21,22], the spectrum of clinical features which characterizes PWS in early life is still relatively unclear or inappropriate molecular testing was performed [21]. In addition, a comparison with healthy infants is, to our knowledge, not yet described. The primary aim of this study was to characterize the clinical features during gestation and in the early life of a large group of children with PWS and to compare these with a birth cohort of healthy children, and with population statistics. By providing an overview of the prenatal and neonatal characteristics of children with PWS, we aim at an earlier recognition of PWS by caregivers. The secondary objective was to assess differences between the three genetic subtypes. We hypothesized that there would be no differences between the genetic subtypes, as we observe no major neonatal differences between the genotypes in our PWS Reference center. However, we expected a higher maternal age in the mUPD group, as the age at which mothers have their children is rising and a higher maternal age increases the risk of mUPD [23].

## 2. Methods

### 2.1. Patients

Patients who were included in the Dutch PWS Cohort Study since the start in 2002 [24] until June 2021, were eligible to be included in the present study. All participants were diagnosed with PWS, confirmed by methylation pattern analysis of the PWS region.

The control group consisted of healthy term-born infants, participating in the Sophia Pluto study (PLUTO cohort), a birth cohort of healthy term-born infants, aiming to provide detailed and longitudinal data on growth and body composition trajectories from birth to age five years [25]. In this PLUTO cohort, most children were included at maternity wards. The prevalence of complications during pregnancy or labor might be higher in the case of hospital delivery since home deliveries are relatively common in the Netherlands. As this may result in bias regarding the pregnancy complications and labor characteristics, we also searched for population statistics in literature to compare the pregnancy and labor characteristics of the PWS group with the (Dutch) general population statistics [26,27,28,29,30,31,32,33,34,35,36,37,38].

### 2.2. Design and Data Collection

All patients were examined during their first visit at the PWS Reference center by the Dutch PWS-team, consisting of a pediatric endocrinologist, a pediatrician, three physicians with PWS experience, a dietician, a psychologist and two nurses. Most data were available from medical records, and both parents and physicians completed standard questionnaires about maternal and perinatal characteristics. The healthy infants were included and examined before the age of 28 days. The Dutch PWS Cohort study (MEC-2001-230, 18 September 2001) and Sophia Pluto Study (MEC-2012-164, 3 November 2012) were approved by the Medical Ethics Committee of the Erasmus University Medical Center. Both studies were conducted according to the guidelines of the Declaration of Helsinki II. Written informed consent was obtained from parents and patients older than 12 years. Assent was obtained from patients younger than 12 years.

Medically assisted reproduction was defined as treatment with intrauterine insemination (IUI), in vitro fertilization (IVF) or intracytoplasmic sperm injection (ICSI). Parents were asked if their child required breathing support after birth (such as CPAP, oxygenation, etc.). Birth weight, -length and -head circumference were converted into Standard Deviation Scores (SDS) with Growth Analyser RCT 4.1, based on Dutch References [39]. Small for gestational age (SGA) was defined as a birth weight below -2 Standard Deviations (SD) [40]. As we have many home deliveries in The Netherlands, birth length was not available in all patients, we, therefore, only used birth weight for the definition of SGA. Apgar scores were considered low when scored below 7 after both 1 and 5 min. Age at diagnosis was defined as the age in weeks at which diagnosis of PWS was genetically confirmed and communicated with the parents.

### 2.3. Statistical Analysis

Statistical analyses were performed using IBM SPSS Statistics 25. Categorical variables were reported using frequencies and continuous variables and were expressed as median ± interquartile range (IQR), as not all variables had a normal distribution. Comparison of variables between the PWS cohort and PLUTO cohort was performed by Mann–Whitney U tests. Categorical data were compared by Chi-Square tests. Differences between the three genetic subtypes were analyzed by Kruskall–Wallis tests. Data of the PWS cohort were compared to the general population (prevalence of variables was derived from literature) using the Z-test for proportion (categorical variables) or the One-Sample Wilcoxon Signed Rank Test (continuous variables). When a range of prevalence was given, the average value was used for statistical analyses. *p*-values of < 0.05 were considered statistically significant.

## 3. Results

### 3.1. Study Group Composition

This study consisted of 244 patients with PWS, including 117 patients (50.0%) with a deletion of the paternal copy of 15q11-13, 106 (43.4%) with mUPD and 10 patients (4.1%) with ICD. In 11 patients (4.5%) the genetic subtype was unknown. The median (IQR) age at the first visit to the Dutch PWS Reference center was 1.23 (0.56; 3.90) years. A group of 365 healthy, term-born infants (PLUTO cohort) was used as a control group. There was no significant difference in sex between the two cohorts or between the genetic subtypes in the PWS study population (Table 1 and Table 2).

### 3.2. PWS Cohort in Comparison with Healthy Children of the PLUTO Cohort

#### 3.2.1. Prenatal Characteristics

Maternal age at delivery tended to be higher in the PWS cohort (median (IQR) 33.0 (30.0–37.0 years) compared to the PLUTO cohort (32.0 (29.0–36.0 years) (*p* = 0.078), and the prevalence of high maternal age at birth (>35 years) was higher in the PWS cohort (*p* = 0.028). The median paternal age did not differ (Table 1). Maternal pre-pregnancy BMI was significantly different between the PWS and PLUTO cohort, with the PWS cohort having more mothers in the BMI range between 20–25 kg/m^2^. There was no difference in parity between the two cohorts. Pregnancy complications did not differ between the two cohorts, except for the amount of amniotic fluid. Both the prevalence of polyhydramnios (27.3% versus 0.5%) and oligohydramnios (7.7% versus 0.3%) were higher in PWS than in the PLUTO cohort. There was no significant difference in miscarriage in maternal history or in use of medically assisted reproduction between the cohorts.

#### 3.2.2. Labor Characteristics

Mode of delivery differed between the cohorts, with a higher caesarean section rate (46.3%) in PWS than in the PLUTO cohort (29.0%), but no difference in the ratio of type 1 (elective) versus type 2 (emergency) caesarean sections (Table 1). A lower rate of assisted vaginal delivery (6.9% versus 12.3%) was found in PWS (*p* <0.001), but the prevalence of breech presentation and induced labor was significantly higher in the PWS (*p* <0.001 and *p* < 0.001, resp).

#### 3.2.3. Birth Measurements

Birth weight and birth length SDS were significantly lower in the PWS compared to the PLUTO cohort (Table 1). Head circumference SDS at birth tended to be higher in PWS (0.74 (−0.11; 1.60) versus 0.53 (−0.53; 1.22) in the PLUTO cohort, resp. (*p* = 0.076)). Infants with PWS were more often SGA compared to the PLUTO cohort (21.1% versus 2.5% (*p* <0.001)).

### 3.3. PWS Cohort in Comparison with the (Dutch) General Population Statistics

#### 3.3.1. Prenatal Characteristics

Maternal age was higher in the PWS cohort in comparison with Dutch population data, 33.0 vs. 30.2 years [26] (*p* < 0.0001), resp. (Table 1). The prevalence of higher maternal age at birth (>35 years) was also higher in PWS, being 40.5% in the PWS cohort and 21.6% in the general population [27] (*p* < 0.001). Paternal age was higher in PWS, compared to the (Dutch) general population (*p* < 0.001). Pregnancy complications occurred more often in PWS, specifically pregnancy hypertension/pre-eclampsia (*p* < 0.001), premature rupture of membranes (*p* <0.001) and both polyhydramnios (*p* < 0.001) and oligohydramnios (*p* < 0.001) [36]. Previous miscarriages also occurred more often in PWS and a trend was found towards a higher prevalence of medically assisted reproduction in PWS (*p* = 0.088) [27].

#### 3.3.2. Labor Characteristics

Both preterm and late/post-term births occurred more often in PWS compared to the (Dutch) general population (Table 1). The caesarean section rate was higher in PWS, being 46.3% in PWS and 17% in the general population [27] (*p* <0.001), without difference in the type of caesarean section. Breech presentation was more common in PWS as well.

### 3.4. Comparison between Genetic PWS Subtypes

#### 3.4.1. Prenatal and Labor Characteristics

In the mUPD group, a higher median maternal age, a higher prevalence of high maternal age at birth (>35 years), and a higher paternal age were found, compared to the other genetic subtypes (Table 2). The difference in age between mothers and fathers is not significantly different between the genetic subtypes (data not shown). No significant differences in maternal pre-pregnancy BMI categories were observed between the genetic subtypes, although there was a tendency towards a lower maternal BMI in the deletion group. There was neither a difference in the prevalence of pregnancy complications, nor in previous miscarriage or medically assisted reproduction between the genetic subtypes. No significant differences were found between the genetic subtypes regarding labor characteristics. Decreased fetal movements during pregnancy were described by 78.5% of the mothers, but there were no differences between the genetic subtypes.

#### 3.4.2. Birth Measurements

No differences were found between the genetic subtypes regarding birth weight, length or head circumference SDS (Table 2). The prevalence of SGA was significantly higher in the mUPD group (31.0%) compared to the deletion (15.2%) and ICD (10.0%) group (*p* = 0.014).

#### 3.4.3. Neonatal Characteristics

Apgar scores did not differ between the genetic subtypes (Table 3). The prevalence of cryptorchidism, breathing problems, tube feeding was also not different between the genetic subtypes. However, the median age in weeks at which the genetic diagnosis of PWS was confirmed, differed significantly between the genetic subtypes (median (IQR): deletion 5.7 (3.0; 12.0) versus mUPD 9.9 (4.5; 26.6)) versus ICD 23.3 (8.2; 59.5)) (*p* < 0.001)).

### 3.5. When to Perform Genetic Testing for PWS in Newborns and Infants

**Figure 1:**
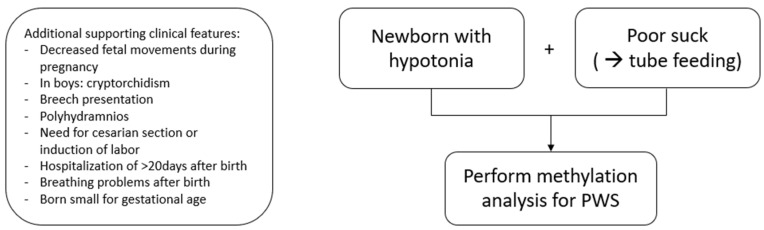
Flowchart for genetic testing and diagnosis of PWS.

Table 4 shows an overview of the most prevalent prenatal and neonatal characteristics of PWS. During pregnancy, 78.5% of the mothers reported decreased fetal movements. In the neonatal period, hypotonia was present in all newborns (100%), and a poor suck led to tube feeding in 93.9%. In boys, cryptorchidism is present in 95.9%. Figure 1 shows a flowchart for genetic testing and diagnosis of PWS and the supporting clinical features.

## 4. Discussion

This study highlights the prenatal and neonatal features in a large cohort of 244 Dutch patients with PWS. We found a higher prevalence of high maternal age at birth (>35 years) and polyhydramnios in the PWS cohort compared to the PLUTO cohort. Compared with general population statistics, pregnancy complications also occurred significantly more often in PWS. Maternal and paternal age were found to be higher in the mUPD group compared to the two other genetic subtypes. Birth weight and length SDS were lower in PWS compared to the PLUTO cohort. Additionally, the prevalence of small for gestational age (SGA) was higher in PWS and particularly in the mUPD group. With regard to delivery characteristics, a higher caesarean section rate, higher prevalence of induction of labor and breech presentation were found in PWS compared to the PLUTO cohort. The neonatal variables were not different between the genetic subtypes, except for the age at genetic diagnosis, which was significantly higher in the mUPD and ICD groups. In addition, we listed the most prevalent perinatal and neonatal characteristics of PWS with the aim to increase the clinical awareness of PWS symptoms and we present a flowchart for genetic testing for PWS in a newborn.

In the PWS cohort, a higher prevalence of mothers being >35 years was found. The highest maternal age was found in the mUPD group, which is in line with a previous study [41]. Advanced maternal age is known to be a risk factor for non-disjunction at meiosis 1, which can lead to trisomic zygotes [42]. Trisomic rescue is an important mechanism in forming a UPD. In these cases, the UPD mechanism begins with two homolog chromosomes who fail to segregate into two, during meiosis. This results in a disomic germ cell, instead of haploid, leading to a trisomic zygote after fertilization. Because most trisomies do not survive, the zygote can rescue itself by losing one of the extra chromosomes. It is expected that this leads to a UPD in 33.3% of cases [43]. Trisomy rescue type mUPD occurs more often in the case of higher maternal age [23]. This can explain the increased prevalence of mUPD over the years [21], as maternal child-bearing age has increased over the years. We also found a higher paternal age in the mUPD group. These findings are in line with other studies [6,18,44,45]. It is yet unclear why a higher paternal age was found, but an explanation could be that it is associated with higher maternal age, as partners are likely to have more or less the same age, which is supported by the finding that there was no significant difference in maternal and paternal age.

Complications were more common during PWS pregnancies, compared to Dutch population statistics, but not compared with the PLUTO cohort. In the PLUTO study, most children were included at maternity wards. Since home deliveries are relatively common in the Netherlands, we expected a higher prevalence of complications during pregnancy and labor in case of hospital deliveries, and, therefore, compared also with Dutch population statistics. Polyhydramnios and a history of miscarriage have been reported as a feature of PWS [6,19,44]. However, reliable population statistics on the prevalence of miscarriage have proven to be difficult, as early miscarriage is often not recognized. The higher prevalence of premature rupture of membranes was reported by Yang et al., but they did not compare the prevalence with the general population [18]. The higher prevalence of pregnancy hypertension, hyperemesis and oligohydramnios has not yet been described. Further investigations of the prevalence and type of pregnancy complications are needed in order to draw definite conclusions.

The prevalence of medically assisted reproduction tended to be higher in PWS compared with the general population. We expected this association to be attributed to increased risk of UPD in pregnancies that resulted from medically assisted reproduction, but there was no difference in the prevalence of medically assisted reproduction between the genetic subtypes. In the literature, an increased risk of imprinting disorders after IVF/ICSI has been described, especially for Angelman and Beckwith-Wiedemann syndrome [46,47,48]. Both these procedures, as well as a defect in the imprinting of the gamete DNA (which had caused infertility), are possible causes of a higher incidence, next to the possibility that parents who are in need of medically assisted reproduction might have an older age.

Notably, birth weight SDS was significantly lower in PWS, while head circumference SDS at birth was not affected. The higher prevalence of SGA birth in the mUPD group has not been reported earlier, although one study found a tendency towards a lower birth weight SDS in the mUPD group [22]. In an Italian cohort of PWS newborns, no difference in birth weight was found between genotypes, but females with a deletion had a smaller birth length compared to the mUPD subtype [20], but we could not confirm this. Asymmetrical fetal growth in terms of an increased head/abdominal circumference ratio has once been reported in fetuses with PWS [49]. The prevalence of placenta abnormalities is unknown, as descriptions of the placenta often lack in medical correspondence, so it might be interesting to investigate this in the future.

The PWS cohort had more preterm (<37 weeks) and late/post-term births (>41 weeks) compared to the general population. The higher rate of preterm births may be explained by the higher prevalence of pregnancy complications, such as decreased fetal movement and preterm rupture of membranes, which increase the risk of induced and premature labor. Post-term delivery may be explained by fetal hypotonia in PWS, which makes spontaneous delivery more difficult. We found no difference in gestational age between the genetic subtypes, which is in line with another recent study [22]. However, the literature is not consistent, as there are studies describing more late/post-term deliveries in mUPD [50] and studies describing more premature births in the mUPD subgroup [41].

Induced labor was more prevalent in PWS than in healthy infants and in the general population. Dudley et al. hypothesized that lower oxytocin levels in PWS infants might be the cause of the higher prevalence of induced labor [44], as oxytocin is known to play a role in parturition. In adults with PWS and mice models, low oxytocin levels have been measured [51,52]. The importance of fetal cortisol in parturition has been discovered a few decades ago [53]. We may hypothesize that a kind of partial secondary fetal adrenal insufficiency [54] may play a role in the difficulties during parturition. However, it is more likely that the hypotonia of the neonate causes most of the induced labors. We did not find higher rates of labor induction in the mUPD group compared to the deletion group, which was previously reported [44]. The rate of caesarean section was found to be higher in PWS infants, as reported earlier [18,19], which may be explained by a number of reasons: the higher prevalence of pregnancy complications, breech presentation, decreased fetal movements and the above-mentioned lack of oxytocin resulting in lack of labor progression. Breech presentation might be the result of fetal hypotonia, which makes moving in the womb difficult.

Next to hypotonia, several neonatal features, such as cryptorchidism and feeding difficulty requiring tube feeding were very common in PWS infants. The prevalence of cryptorchidism was similar as reported in other studies [6,19,45,55]. The prevalence of tube feeding was higher in our cohort compared to other reports [6,18,45,55]. The rate of breathing problems was difficult to compare with other studies as different definitions have been used (e.g., only CPAP use or nasal oxygenation).

The age at genetic diagnosis was significantly higher in the mUPD and the ICD groups, which is in line with a recent study, describing an earlier diagnosis in patients with a deletion [22]. As an explanation, they hypothesized that the manifestation of PWS is more evident in newborns with a deletion, but they had no data about hypotonia or feeding difficulties to support their hypothesis. As we found no differences in neonatal variables between the genetic subtypes, we hypothesize that the difference in time until the diagnosis is dependent on the method of testing. A deletion will be found with an SNP array, whereas mUPD would only show up if there are homozygotic regions due to isodisomy and the ICD subtype cannot be found with an SNP array. Bar et al. also stated that if inappropriate molecular tests are used, false negative results can occur [21]. We, therefore, advise performing methylation analysis if PWS is suspected, as this is the recommended test to diagnose PWS [56].

Since the development of methylation analysis, the diagnosis of PWS can be made with 100% certainty. Diagnosis is nowadays increasingly suspected in the first weeks of life, with a genetic diagnosis a few weeks later. However, in many countries and also in our PWS cohort, delayed diagnoses are present when symptoms are not recognized or milder than expected with PWS (e.g., no need for tube feeding). No single prenatal or neonatal finding is specific for PWS, but PWS should be considered when a newborn or infant has hypotonia in combination with a poor suck and tube feeding, particularly in the case of severely decreased fetal movements. Next to these core features, there are supportive clinical features, such as cryptorchidism in boys, polyhydramnios, breech presentation, induction of labor, caesarean section, born SGA, breathing problems and prolonged hospitalization (>20 days). In literature, hypotonia is often described as central or peripheral [57,58], but this distinction is not always clear for clinicians [58]. The presence of facial dysmorphisms is often part of the algorithm for hypotonia [57,58], but in our experience, dysmorphisms in newborns with PWS are often subtle. Therefore, our advice is to perform genetic testing for PWS if a newborn presents with hypotonia and poor suck.

Our study has several strengths, such as the large number of patients with PWS. As the Dutch PWS Cohort study has been following patients since 2002, it was possible to include many young PWS patients over the years. Our study has more patients with mUPD compared to other studies [4,6,18,44], which enabled a better comparison between genetic subtypes. Another strength is the large amount of prospective data. By comparing the PWS cohort to both the PLUTO cohort and the (Dutch) general population, we have tried to ensure that our findings show the characteristics through which infants with PWS can be suspected prenatally and recognized soon after birth. This is the first study that statistically analyzed the differences between PWS infants and healthy infants and Dutch population statistics.

Most of the characteristics were in medical records or asked and recorded by the PWS team during the first visit at the PWS Reference center when the infants had a median age of 1.23 years. The questions about the perinatal period were, however, prone to some recall bias. Another limitation is the potential bias of the PLUTO study, which included most infants at maternity wards. Since home deliveries are relatively common in the Netherlands, the prevalence of complications during pregnancy or labor might be higher in hospital deliveries, which may result in bias regarding the pregnancy complications and labor characteristics in the PLUTO cohort. We, therefore, added the comparison with the general population statistics.

In conclusion, our study presents an overview of prenatal and neonatal variables in infants with PWS compared to healthy infants. Our findings show that suspicion of PWS can be raised prenatally and postnatally and that the diagnosis can be made within the first weeks of life. When the prenatal PWS phenotype is recognized by obstetricians, this can prepare both obstetricians and families. The recognition of early symptoms in newborns by clinicians and other health care workers is important for early diagnosis and the start of multidisciplinary treatment, which can lead to better outcomes in later life. Our findings may increase clinical awareness of the early perinatal signs of PWS.

## Figures and Tables

**Table 1:** Characteristics of the PWS cohort compared to the PLUTO cohort and population statistics.

	PWS Cohort (*n* = 244)	PLUTO Cohort (*n* = 365)	*p*-Value *	Population Statistics	*p*-Value **
**Maternal and Pregnancy Characteristics**					
Maternal age (years)	33.0 (30.0; 37.0)	32.0 (29.0; 36.0)	0.078	30.2 [26]	<0.001
High maternal age (>35 years)	92 (40.5)	115 (31.7)	0.028	21.6 [27]	<0.001
Paternal age (years)	34.0 (32.0; 39.0)	34.0 (31.0; 38.0)	0.112	33.0 [28]	<0.001
High paternal age (>35 years)	108 (49.1)	137 (45.1)	0.362	NF	NA
Maternal pre-pregnancy BMI (kg/m^2^)			0.008	NF	NA
BMI < 20	7 (5.8)	39 (11.4)			
BMI 20–25	86 (71.7)	188 (55.0)			
BMI 25–30	23 (19.2)	78 (22.8)			
BMI 30–35	4 (3.3)	25 (7.3)			
BMI > 35	0 (0.0)	12 (3.5)			
Parity	1.0 (1.0; 2.0)	1.0 (1.0; 2.0)	0.599	NF	NA
History of miscarriage	50 (22.0)	88 (24.1)	0.560	15–20 [29]	0.044
Medically assisted reproduction	10 (4.5)	25 (6.8)	0.250	2.8 [27]	0.088
Hypertension/pre-eclampsia	13 (5.8)	30 (8.2)	0.075	1–3 [30]	<0.001
Gestational diabetes	6 (2.7)	14 (3.8)	0.457	2–5 [31]	0.317
Premature rupture of membranes	19 (8.5)	19 (5.2)	0.113	1–8 [32]	<0.001
Hyperemesis	14 (6.3)	19 (5.2)	0.584	0.3–3 [33]	<0.001
Amniotic fluid					
Polyhydramnios	57 (27.3)	2 (0.5)	<0.001	0.2–3.9 [34,35]	<0.001
Oligohydramnios	16 (7.7)	1 (0.3)	NA	<1.0–4.4 [36]	<0.001
**Labor Characteristics**					
Term			<0.001 ^#^		
Preterm <37 weeks	42 (17.6)	^#^		7.9 [27]	<0.001
Full term 37–41 weeks	131 (54.8)	316 (86.6)		74.4	<0.001
Late/Post-term >41 weeks	66 (27.6)	49 (13.4)		17.7 [37]	<0.001
Mode of delivery			<0.001		
Vaginal delivery	102 (46.8)	212 (58.6)		73 [27]	<0.001
Assisted delivery	15 (6.9)	45 (12.4)		10 [27]	0.418
Caesarean section (CS)	101 (46.3)	105 (29.0)		17 [27]	<0.001
Type of CS			0.594		0.260
Primary	40 (41.2)	45 (45.0)		45.0 [27]	
Secondary	57 (58.8)	55 (55.0)		55.0 [27]	
Induction of labor	86 (38.6)	44 (12.1)	<0.001	21.4 [27]	<0.001
Breech presentation	70 (31.0)	17 (4.7)	<0.001	3–4 [38]	<0.001
**Birth Measurements**					
Male	130 (53.3)	210 (57.5)	0.300	NF	NA
Birth weight SDS	−1.06 (−1.78; −0.34)	0.26 (−0.49; 1.00)	<0.001	NF	NA
Birth length SDS	−0.27 (−1.32; 0.72)	0.73 (−0.18; 1.45)	<0.001	NF	NA
Birth head circumference SDS	0.74 (−0.11; 1.60)	0.53 (−0.53; 1.22)	0.076	NF	NA
Small for gestational age	50 (21.1)	9 (2.5)	<0.001	NF	NA

Data are expressed as median (IQR) or n (%). NF = not found NA = not applicable. * *p*-Value between PWS and PLUTO cohort. ** *p*-Value between PWS cohort and population statistics ^#^ Full term birth (gestational age of 37 weeks or more) was one of the inclusion criteria of the PLUTO cohort.

**Table 2:** Characteristics of the PWS genetic subtypes.

	Deletion (*n* = 117)	mUPD (*n* = 106)	ICD (*n* = 10)	*p*-Value
**Maternal and Pregnancy Characteristics**				
Maternal age (years)	31.0 (28.0; 34.0)	36.0 (32.8; 39.0)	28.0 (24.0; 33.5)	<0.001
High maternal age (>35 years)	25 (22.5)	60 (61.2)	2 (22.2)	<0.001
Paternal age (years)	33.0 (30.3; 36.0)	37.0 (33.0; 40.0)	30.5 (28.3; 40.0)	<0.001
High paternal age (>35 years)	36 (33.3)	65 (67.7)	2 (25.0)	<0.001
Maternal pre-pregnancy BMI (kg/m^2^)				0.043
BMI < 20	6 (9.5)	1 (1.9)	0 (0.0)	
BMI 20–25	43 (68.3)	39 (75.0)	3 (75.0)	
BMI 25–30	14 (22.2)	9 (17.3)	0 (0.0)	
BMI 30–35	0 (0.0)	3 (5.8)	1 (25.0)	
BMI > 35	0 (0.0)	0 (0.0)	0 (0.0)	
Parity	1 (1.0; 2.0)	1 (1.0;2.0)	2 (1.0;2.3)	0.967
History of miscarriage	27 (24.1)	22 (22.7)	0 (0.0)	0.249
Medically assisted reproduction	5 (4.6)	3 (3.2)	1 (11.1)	0.514
Hypertension/pre-eclampsia	6 (5.1)	4 (4.3)	1 (11.1)	0.771
Gestational diabetes	1 (0.9)	5 (5.3)	0 (0.0)	0.141
Premature rupture of membranes	9 (8.1)	8 (8.5)	1 (11.1)	0.951
Hyperemesis	6 (5.4)	7 (7.4)	1 (11.1)	0.716
Amniotic fluid				
Polyhydramnios	27 (25.5)	26 (30.6)	2 (22.2)	0.687
Oligohydramnios	7 (6.6)	6 (7.1)	2 (22.2)	0.228
Decreased fetal movements	71 (73.2)	72 (83.7)	3 (60.0)	0.147
**Labor Characteristics**				
Term (weeks)				0.183
Preterm <37	15 (13.0)	22 (21.2)	3 (30.0)	
Full term 37–41	63 (54.8)	58 (55.8)	3 (30.0)	
Late/Post-term >41	37 (32.2)	24 (23.1)	4 (40.0)	
Mode of delivery				0.258
Vaginal delivery	54 (50.0)	39 (41.9)	6 (75.0)	
Assisted delivery	8 (7.4)	6 (6.5)	1 (12.5)	
Caesarean section	46 (42.6)	48 (51.6)	1 (12.5)	
Type of caesarean section				0.487
Primary	18 (40.9)	19 (40.4)	1 (100)	
Secondary	26 (59.1)	28 (59.6)	0 (0.0)	
Induction of labor	43 (39.1)	35 (36.8)	3 (33.3)	0.909
Breech presentation	34 (30.6)	32 (33.0)	1 (11.1%	0.703
**Birth Characteristics**				
Male	64 (54.7)	57 (53.8)	7 (70.0)	0.320
Birth weight SDS	−1.16 (−1.71; −0.33)	−1.09 (−2.10; −0.41)	−0.30 (−1.34; 0.03)	0.220
Birth length SDS	−0.47 (−1.47; 0.70)	−0.24 (−1.06; 0.86)	0.15 (−0.21; 0.81)	0.505
Birth head circumference SDS	0.95 (0.11; 1.65)	0.52 (−0.13; 1.36)	1.08 (0.01; 2.73)	0.171
Small for gestational age	17 (14.8)	31 (30.4)	1 (10.0)	0.014

Data are expressed as median (IQR) or *n* (%).

**Table 3:** Neonatal characteristics of the PWS cohort and between the different genotypes.

	PWS Cohort (*n* = 244)	Deletion (*n* = 117)	mUPD (*n* = 106)	ICD (*n* = 10)	*p*-Value *
Apgar score at 1 min	7.0 (5.0; 8.0)	7.0 (5.0; 8.0)	7.0 (5.0; 8.0)	6.5 (5.8; 8.3)	0.998
Low Apgar score at 1 min	55 (40.7)	25 (41.0)	25 (39.7)	3 (50.0)	0.885
Apgar score at 5 min	8.0 (7.0; 9.0)	9.0 (7.5; 9.0)	8.0 (7.0; 9.0)	8.5 (6.0; 9.0)	0.642
Low Apgar score at 5 min	14 (10.1)	5 (8.2)	7 (10.8)	2 (33.3)	0.162
Cryptorchidism (boys)	118 (95.9)	59 (96.7)	51 (94.4)	3 (100)	0.769
Hypotonia	244 (100.0)	117 (100.0)	106 (100.0)	10 (100.0)	NA
Breathing problems	116 (64.4)	56 (65.9)	53 (65.4)	5 (62.5)	0.981
Poor sucking leading to tube feeding	216 (93.9)	106 (94.6)	93 (93.0)	9 (100)	0.656
Tube feeding duration (weeks)	15.0 (6.0; 25.0)	16.0 (6.0; 24.0)	12.5 (6.0; 24.8)	19.5 (4.4; 41.0)	0.667
Duration of hospitalization (days)	24.0 (16.0; 35.0)	24.5 (17.0; 35.3)	22.5 (14.3; 35.0)	25.0 (20.0; 31.0)	0.733
Age at diagnosis (weeks)	7.9 (3.8; 22.8)	5.7 (3.0; 12.0)	9.9 (4.5; 26.6)	23.3 (8.2; 59.5)	0.001

Data are expressed as median (IQR) or n (valid%). NA = not applicable. * *p*-Value between the genetic subtypes.

**Table 4:** Characteristics of Prader-Willi Syndrome during pregnancy and neonatal period.

Characteristics During Pregnancy	Prevalence
Decreased fetal movements	78.5%
Caesarian section	46.3%
Induction of labor	38.6%
Breech presentation	31.0%
Polyhydramnios	27.3%
**Characteristics During the Neonatal Period**	**Prevalence**
Hypotonia	100%
Poor suck leading to tube feeding	93.9%
Cryptorchidism in boys	95.9%
Hospitalization of > 20 days after birth	65.9%
Breathing problems after birth	64.4%
Small for gestational age	21.1%

## Data Availability

The datasets generated during and/or analyzed during the current study are not publicly available but are available from the corresponding author on reasonable request.
